# Safety and efficacy of plasma exchange treatment in children with AQP4-IgG positive neuromyelitis optica spectrum disorder

**DOI:** 10.3389/fimmu.2022.1113406

**Published:** 2023-01-05

**Authors:** Zhichao Li, Lin Wan, Xinting Liu, Jing Wang, Xiuyu Shi, Huanfen Zhou, Quangang Xu, Shihui Wei, Guang Yang

**Affiliations:** ^1^ Medical School of Chinese People’s Liberation Army, Beijing, China; ^2^ Department of Pediatrics, The First Medical Center, Chinese People’s Liberation Army General Hospital, Beijing, China; ^3^ Senior Department of Pediatrics, The Seventh Medical Center of PLA General Hospital, Beijing, China; ^4^ Department of Ophthalmology, The First Medical Center, Chinese People’s Liberation Army General Hospital, Beijing, China; ^5^ The Second School of Clinical Medicine, Southern Medical University, Guangzhou, China

**Keywords:** neuromyelitis optica spectrum disorders, children, plasma exchange, AQP4-IgG, prognosis

## Abstract

Neuromyelitis optica spectrum disorder (NMOSD), a severe demyelinating disease, is rare among children. Plasma exchange (PE) is widely used as a salvage therapy for severe and corticosteroid-unresponsive patients with NMOSD. Presently, there are limited studies on the safety and efficacy of PE in children with NMOSD. Herein, we report the case of six children with NMOSD who received PE along with the outcomes and adverse events. All six children (female, age at onset 4 years 9 months–13 years 2 months) were AQP4-IgG positive and received standard PE using the COM.TEC Cell Separator. The interval between NMOSD onset and PE was 29 days (range 10–98). Only one patient (P3) who received PE 10 days after acute exacerbations exhibited clinical improvement. Her left visual acuity increased from 0.06 to 0.6 (spectacle-corrected visual acuity was 1.0) and her EDSS score decreased from 4 to 3 points. The other five patients had no clinical improvement and no EDSS scores changes after PE. Adverse events included rashes (P1, P3), acute non-occlusive thrombosis of the internal jugular vein (P1), and thrombocytopenia (P2). In conclusion, the timing of PE initiation as a rescue therapy for severe and corticosteroid-unresponsive pediatric AQP4-IgG positive NMOSD may be crucial to treatment efficacy, and early initiation of PE may be associated with a better outcome. Furthermore, PE has the potential risk for clinically significant adverse effects that should be considered before initiating the therapy and should be weighed against potential benefits.

## Introduction

NMOSD is a central nervous system (CNS) inflammatory syndrome characterized by immune-mediated demyelination and axonal destruction, mostly involving the optic nerves and the spinal cord (1). The presence of circulating water channel protein aquaporin 4 (AQP4) IgG distinguishes NMOSD from classic multiple sclerosis (MS) (2). Recently,myelin oligodendrocyte glycoprotein (MOG) were detected in those AQP4-IgG-seronegative NMOSD patients. In children, AQP4-IgG-NMOSD are rarer than MOG-IgG-NMOSD. Patients with AQP4-IgG showed a poor prognosis than those with MOG-IgG, including more attacks and poor recovery (3). AQP4 is widely distributed in the astrocytic foot processes at the blood-brain barrier and is particularly concentrated in the grey matter, periaqueductal, and periventricular regions of the spinal cord (4). AQP4 IgG binds to water channels present on astrocytes, which trIgGers an immune response leading to complement activation and quick destruction of astrocytes and neurons and causing inflammation and demyelination of the optic nerve and spinal cord (5). The risk of NMOSD is higher in Asians than in Westerners (6).

Approximately 90% of NMOSD cases have a relapsing course (7). Typically, substantial residual deficits follow the initial and subsequent assaults, resulting in the rapid development of blindness and paraplegia within five years (8). The high rate of disability among patients with NMOSD makes therapy during acute attacks crucial for minimizing irreversible damage to the CNS. High-dose steroids are the first-line treatment during acute attacks (9). Methylprednisolone (MP) is the most commonly used steroid. For severe neurological deficits and corticosteroid-unresponsive patients, apheresis therapies are alternative treatment (10). Apheresis therapies are treatment *via* eliminate pathogenic antibodies and other proinflammatory factors from the patient’s circulation, which include two major techniques (plasma exchange,PE and immunoadsorption, IA) (11). PE showed robust efficacy in pediatric demyelination including NMOSD (12).

For the patients with NMOSD, it seems that early PE was more effective. Complete remission of symptom was reached more often with early PE (start after symptom onset day 0–6: 29%; day ≥7: 3.7% than with early IA treatment (start after symptom onset day 0–6: 6.7%; day ≥7: 0%) (13).

NMOSD is more prevalent in adults and few cases have been described in children. At present, there are few studies on the safety and efficacy of PE for pediatric NMOSD. Herein, we report our experience with PE in children with NMOSD at a single center.

## Materials and methods

### Study subjects

Six pediatric patients with NMOSD who were referred to the First Medical Center of Chinese PLA General Hospital between June 2015 and June 2022 and underwent PE were enrolled. The most recent criteria were used for NMOSD diagnosis (1).

### Laboratory analysis

The patients’ serum and cerebrospinal fluid (CSF) were collected before PE treatment for analysis. CSF test included total CSF cell counts, white blood cell (WBC) count, total protein level, glucose level, IgG index (the normal IgG index reference was <0.65), and oligoclonal band (positive as 2 or more bands were present in the CSF but not in the corresponding serum). Serum test included antinuclear antibody (ANA) titers, autoantibodies against double stranded DNA, Sjögren syndrome A (SSA)/B (SSB), ribosomal p protein, Scl-70, Jo-1, thyroglobulin (TG), thyroid peroxidase (TPO), β2-glycoprotein I antigen, AQP4-IgG and MOG-IgG. As the main assessment component, the AQP4-IgG was test by the cell-based assays (CBA), as previously reported, using HEK-293 cells stably transfected with the M23 isoform of AQP4 (14). A twofold dilution (1:80, 1:160, 1:320, 1:640, 1:1280) was used to create endpoint titers. The MOG-IgG was detected by Euroimmun fixed CBA as reported (15).

### Clinical assessment, NMOSD diagnosis, and PE initiation

Complete blood count, prothrombin time, fibrinogen levels, partial thromboplastin time, and comprehensive metabolic panel were evaluated before PE initiation. The transfusion department assessed the children’s vascular condition and lab results before deciding which vascular access to use and the total amount of plasma to be exchanged. The volume of plasma replacement and frequency and number of procedures were based on the American Society for Apheresis guidelines (11). Considering the body weight and physical condition, the total amount of plasma exchange is typically 40 mL/kg each cycle. COM.TEC Cell Separators (Fresenius Healthcare) were used for the PE procedure. PE was performed every alternate day for five cycles. We performed plasma exchange with 5% albumin (ALB). Plasma was removed during the PE process, and fresh frozen plasma (FFP) or/and frozen plasma (FP) along with 0.9% sodium chloride solution, albumin, acid-citrate-dextrose (ACD) anticoagulant, and calcium gluconate solution was infused as a substitution. The plasma exchange procedures of six patients are summarized in **Table 1**.

**Table 1:** PE procedure of patients with NMOSD.

NO.	P1	P2	P3	P4	P5	P6
Cycles	5	2	5	5	5	5
Body weight	30.6Kg	49Kg	40Kg	64Kg	62Kg	66Kg
Blood type	B; RH+	A; RH+	AB; RH+	O; RH+	B; RH+	O; RH+
Interval time of each cycle	1 day	1 day	1 day	1 day	1 day	1 day
Cycle 1	ACD 164ml; ALB 20g + NS 250ml; NS 220ml; CG 2g; FFP 981ml	ACD 227ml; ALB 20g+ NS 250ml; NS 370ml; CG 4g; FFP 1350ml; GS 250ml	ACD 198ml; ALB 20g+ NS 250ml; NS 350ml; CG 6g; FFP 1410ml	ACD 253ml; ALB 20g+ NS 250ml; NS 1150ml; CG 4g; FFP 1899ml	ACD 248ml; ALB 20g+ NS 250ml; NS 1150ml; CG 6g; FFP 2000ml	ACD 261ml; ALB 20g+ NS 250ml; NS 1250ml; CG 4g; FFP 1827ml
Cycle 2	ACD 155ml; ALB 20g+ NS 250ml; NS 200ml CG 2g; FFP 738ml; GS 250ml	ACD 227ml; ALB 20g+ NS 250ml; NS 420ml; CG 4g; FP 945ml FFP 432ml; GS 250ml	ACD 189ml; ALB 20g+ NS 250ml; NS 300ml; CG 6g; FFP 1480ml	ACD 256ml; ALB 20g+ NS 250ml; NS 1150ml; CG 4g; FFP 1890ml	ACD 257ml; ALB 20g+ NS 250ml; NS 700ml; CG 6g; FFP 1800ml	ACD 258ml; ALB 20g+ NS 250ml; NS 1250ml; CG 4g; FFP 1800ml
Cycle 3	ACD 163ml; ALB 20g + NS 250ml; NS 520ml; CG 2g; FFP 729ml		ACD 205ml; ALB 20g+ NS 250ml; NS 320ml; CG 6g; FFP 1520ml	ACD 252ml; ALB 20g+ NS 250ml; NS 1150ml; CG 4g; FFP 1350ml	ACD 248ml; ALB 20g+ NS 250ml; NS 1150ml; CG 6g; FFP 2300ml	ACD 264ml; ALB 20g+ NS 250ml; NS 1250ml; CG 4g; FFP 1917ml
Cycle 4	ACD 164ml; ALB 20g + NS 250ml; NS 420ml CG 4g; FFP 729ml		ACD 195ml; ALB 20g+ NS 250ml; NS 230ml; CG 6g; FFP 1520ml; GS 500ml	ACD 258ml; ALB 20g+ NS 250ml; NS 1150ml; CG 4g; FFP 1980ml	ACD 253ml; ALB 20g+ NS 250ml; NS 1150ml; CG 6g; FFP 1700ml	ACD 257ml; ALB 20g+ NS 250ml; NS 1250ml; CG 4g; FFP 1809ml
Cycle 5	ACD 157ml; ALB 20g + NS 250ml; NS 370ml CG 2g; FFP 909ml		ACD 196ml; ALB 20g+ NS 250ml; NS 750ml; CG 6g; FFP 1480ml	ACD 255ml; ALB 20g+ NS 250ml; NS 11500ml; CG 4g; FFP 1728ml	ACD 253ml; ALB 20g+ NS 250ml; NS 700ml; CG 6g; FFP 1800ml	ACD 263ml; ALB 20g+ NS 250ml; NS 1250ml; CG 4g; FFP 1863ml

ACD, acid-citrate-dextrose; ALB, albumin; CG, calcium gluconate; FP, frozen plasma; FFP, fresh frozen plasma; GS, glucose solution; NS, Normal saline solution (0.9% sodium chloride solution).

By reviewing the medical charts, detailed clinical information was collected including age at onset, clinical manifestations, magnetic resonance imaging (MRI), physical examination, delay in PE initiation, acute phase and maintenance immunosuppressive therapy, time to first relapse after PE, and EDSS scores during nadir, and before and after PE procedures. Adverse events associated with the PE procedure were recorded such as hypotension, nausea, hypocalcemia, hypofibrinogenemia, thrombocytopenia, and acute non-occlusive thrombosis of the internal jugular vein.

## Results

### Case 1

A 8-year 3-month-old girl was admitted to the local hospital because of progressive vision loss in the left eye with visual field defects appearing one week before admission. Her right visual acuity was 1.2 and her left visual acuity was hand movements (HM) at 10 cm with no improvement after correction. Lab tests revealed the presence of AQP4-IgG, antinuclear antibodies (ANA), rheumatoid factor, and HLA-B27. MRI results showed a slight thickening of the left optic nerve with significant enhancement; thus, optic neuritis was considered. After receiving intravenous methylprednisolone (IVMP) and intravenous immunoglobulins (IVIG), the patient’s left visual field deficit improved but there was no improvement in vision. Then oral MP was given as maintenance therapy. The patient was then admitted to our hospital, and five cycles of PE procedures were performed. However, the child’s visual acuity did not improve at the time of discharge. Adverse events included rashes and acute non-occlusive thrombosis of the internal jugular vein. The patient received oral MP, mycophenolate mofetil (MMF), and aspirin treatment after being discharged from the hospital. After PE, the patient suffered 3 relapses within 47 months of follow-up. The duration between the acute attack and the PE procedure was 98 days while that between admission and the PE procedure was 91 days (**Table 2**).

**Table 2:** Clinical phenotype of patients with NMOSD.

NO.	P1	P2	P3	P4	P5	P6
Sex	Female	Female	Female	Female	Female	Female
Age at first attack	8 years 3 months	12 years 7 months	10 years 9 months	13 years 2 months	10 years	12 years 2 months
Phenotype at first attack	Left ON	Myelitis	Right ON	Left ON	Bilateral ON	Left ON
Brain MRI	Normal	T1-weighted and T2-weighted imaging signals in the bilateral centrum semiovale and periventricular white matter	Normal	Normal	Normal	Normal
ON MRI	slight thickening of the left optic nerve with significant enhancement	long T2 and T1-weighted imaging enhancement in the retrobulbar and intraorbital of right optic nerve	bilateral abnormal optic nerve signal with slight enhancement	long T2-weighted imaging with enhancement in the left intraorbital optic nerve	NA	long T2-weighted and T1-weighted imaging enhancement in the retrobulbar of right optic nerve
Spinal MRI lesion	Normal	NA	NA	NA	abnormal signal in L2-L3	NA
Serum AQP4-IgG titer before PE	1:160	1:320	1:640	1:320	1:320	1:640
MOG-IgG status	Negative	Negative	Negative	Negative	Negative	Negative
ANA	Positive	Positive	Negative	Negative	Negative	Positive
CSF cell counts	NA	131/µl	0/µl	NA	3/µl	4/µl
CSF OCB	Negative	Negative	Negative	Negative	Negative	Negative
CSF IgG	NA	2.68mg/dl	0.867mg/dl	NA	1.5mg/dl	1.54mg/dl
CSF albumin	NA	29.2mg/dl	17.6mg/dl	NA	28.9mg/dl	26.6mg/dl
CSF IgG index	NA	0.354	0.225	NA	0.445	0.321
Number of attacks before PE	1	3	2	2	3	2
Number of total attacks	3	3	2	2	3	3
Time from acute attack to PE	98 days	40 days	10 days	28 days	30 days	17 days
Time from admission to PE	91 days	15 days	3 days	8 days	11 days	8 days
Treatment before PE	IVMP+ IVIG	IVMP	IVMP	IVMP	IVMP	IVMP
Attacked eye before PE	OS	OS	OS	OS	OS	OS
Visual acuity during nair(corrected)	HM 10CM (HM 10CM)	NLP	0.06(0.06)	HM 5CM (HM 5CM)	HM 5CM (HM CM)	NLP
Visual acuity before PE(corrected)	HM 40CM (HM 40CM)	0.04(0.04)	0.1(0.1)	HM 5CM (HM 5CM)	HM 5CM (HM CM)	HM 40CM (HM 40CM)
Visual acuity after PE(corrected)	HM 40CM (HM 40CM)	0.06(0.06)	0.6(1.0)	CF 50CM (CF 50CM)	HM 5CM (HM CM)	0.07(0.07)

ANA, antinuclear antibody; CF, counting fingers; CSF, cerebrospinal fluid; HM, hand movements; IVIG, intravenous immunoglobulins; IVMP, intravenous methylprednisolone; NLP, no light perception; OCB, oligoclonal band; OD, oculus dexter; OS, oculus sinister; PE, Plasma exchange; NA, not applicable; ON, optic neuritis.

### Case 2

A 12-year 7-month-old girl was admitted to the local hospital due to a 2-day history of numbness and immobility of the extremities, accompanied by vomiting and urinary retention. MRI showed high T1- and T2-weighted signals in the bilateral centrum semiovale and periventricular white matter. After receiving IVMP, the patient’s symptoms progressively improved. Then oral MP was given as maintenance therapy. Six months later, the patient was admitted to the Pediatric Department again because of vision loss in the right eye and ocular pain. MRI revealed long T2- and T1-weighted signal enhancement in the retrobulbar and intraorbital of the right optic nerve. The patient recovered after receiving IVIP. Five months after the second attack, the patient was admitted to our hospital due to loss of vision in the left eye and ocular pain. Her right visual acuity was 0.15 with no improvement after correction, while the left eye exhibited loss of light perception. Lab tests revealed the presence of AQP4-IgG, ANA, anti-SSA antibodies, and anti-Ro-52 antibodies. Following high-dose IVMP, the patient’s vision did not improve, and plasma exchange was initiated. PE treatment was stopped after two cycles due to the development of severe thrombocytopenia. After the PE therapy, the patient regained light perception in the right eye, but the vision remained poor. Rituximab (RTX) was used to prevent relapse. During the 45 months of follow-up, no relapse was observed. The duration between the acute attack and PE initiation was 40 days, while the duration between admission and PE initiation was 15 days (**Table 2**).

### Case 3

A 10-year 9-month-old girl was admitted to the local hospital because of vision loss in the right eye, ocular pain, and headache. The right eye exhibited loss of light perception and the left visual acuity was 1.5. After receiving IVMP, ocular pain and headache improved and the light perception of the right eye recovered, however, vision remained poor. Then oral MP was given as maintenance therapy. After 27 months, the patient was admitted to the local hospital again because of vision loss in the left eye and ocular pain. Her right visual acuity was 0.02 and her left visual acuity was 0.06 with no improvements after correction. Following high-dose IVMP, the patient’s vision did not improve and she was admitted to our hospital. Lab tests were positive for AQP4-IgG. MRI revealed abnormal and slightly enhanced signals from the bilateral optic nerve. The patient received five cycles of PE without any significant adverse events, except for rashes that were visible on day 1. Following PE, the left visual acuity increased to 0.6 (spectacle-corrected visual acuity was 1.0) at the time of discharge. RTX was used to prevent relapse. During the 81 months of follow-up, no recurrence was observed. The duration between the acute attack and PE initiation was 10 days while that between admission and PE initiation was 3 days (**Table 2**).

### Case 4

A 13-year 2-month-old girl was admitted to the local hospital because of vision loss and ocular pain in the left eye. Her right visual acuity was 0.1 (spectacle-corrected visual acuity was 1.0) and her left visual acuity was counting fingers (CF) at 20 cm with no improvement after correction. After receiving IVMP, ocular pain progressively improved, and the spectacle-corrected visual acuity of the left eye improved to 0.3. Then oral MP was given as maintenance therapy. Two weeks later, the patient was admitted to the local hospital again due to a decrease in visual acuity (0.02) in the left eye. Lab tests were positive for AQP4-IgG. MRI revealed long T2-weighted signals with enhancement in the left intraorbital optic nerve. Following high-dose IVMP, the patient’s vision did not improve. The patient was then admitted to our hospital, and five cycles of PE procedures were performed without any adverse events. Following PE, the child’s visual acuity did not improve at the time of discharge. RTX was used to prevent relapse. During the 34 months of follow-up, no recurrence was observed. The duration between the acute attack and PE initiation was 28 days while that between admission and PE initiation was 8 days (**Table 2**).

### Case 5

A 10-year-old girl was admitted to the local hospital because of vision loss and loss of light perception in bilateral eyes. After receiving IVMP, the left visual acuity recovered while the right visual acuity showed no improvement. Then oral MP was given as maintenance therapy. After 24 months, the patient was readmitted to the local hospital because of numbness in the right upper extremity with limb weakness. MRI revealed abnormal signals from L2-L3. Following high-dose IVMP, the patient’s symptoms progressively remitted. Ten months after the second attack, the patient was again admitted to the local hospital because of vision loss in the left eye with visual field defects. Following high-dose IVMP, the patient’s vision did not improve this time, and she was admitted to our hospital. Lab tests were positive for AQP4-IgG. Five cycles of PE procedures were performed without any adverse events. The child’s visual acuity showed no improvement at the time of discharge. Azathioprine (AZA) was used to prevent relapse. During the 33 months of follow-up, no recurrence occurred. The duration between the acute attack and PE initiation was 30 days while that between admission and PE initiation was 11 days (**Table 2**).

### Case 6

A 12-year 2-month-old girl was admitted to the local hospital because of vision loss and ocular pain in the right eye. Her right eye exhibited loss of light perception and her left visual acuity was 1.0. The patient was then admitted to our hospital. After receiving IVMP, ocular pain progressively remitted, and light perception of the right eye improved, but the vision remained poor. Then oral MP was given as maintenance therapy. Lab tests revealed AQP4-IgG and ANA positivity. MRI revealed long T2- and T1-weighted enhancements in the retrobulbar of the right optic nerve. After receiving IVMP, ocular pain improved progressively, and light perception of the right eye recovered, however, vision remained poor. Five months later, the patient was readmitted to our hospital because of vision loss and ocular pain in the left eye. Her right visual acuity was 0.05 with no light perception in the right eye. MRI revealed abnormal signal enhancement of the left optic nerve. Following high-dose IVMP, ocular pain showed progressive remission, and light perception of the left eye recovered, but the vision remained poor. Five cycles of PE procedures were performed without adverse events; however, the child’s visual acuity did not improve. RTX was used to prevent relapse. After PE, the patient suffered a relapse within 40 months of follow-up. The duration between the acute attack and PE was 17 days while that between admission and PE was 8 days (**Table 2**).

### Treatment and outcome

All six patients received IVMP at an initial dose of 10–20 mg/day after the acute attack. PE was performed when the patient’s symptoms did not considerably improve after administering high doses of steroids. The median duration between the acute event onset and PE initiation was 29 days (range 10–98). Five patients (P1, P3-P6) received five rounds of PE while one patient (P2) underwent two rounds of PE before stopping the procedure due to severe thrombocytopenia (**Table 2**).

**Figure 1:**
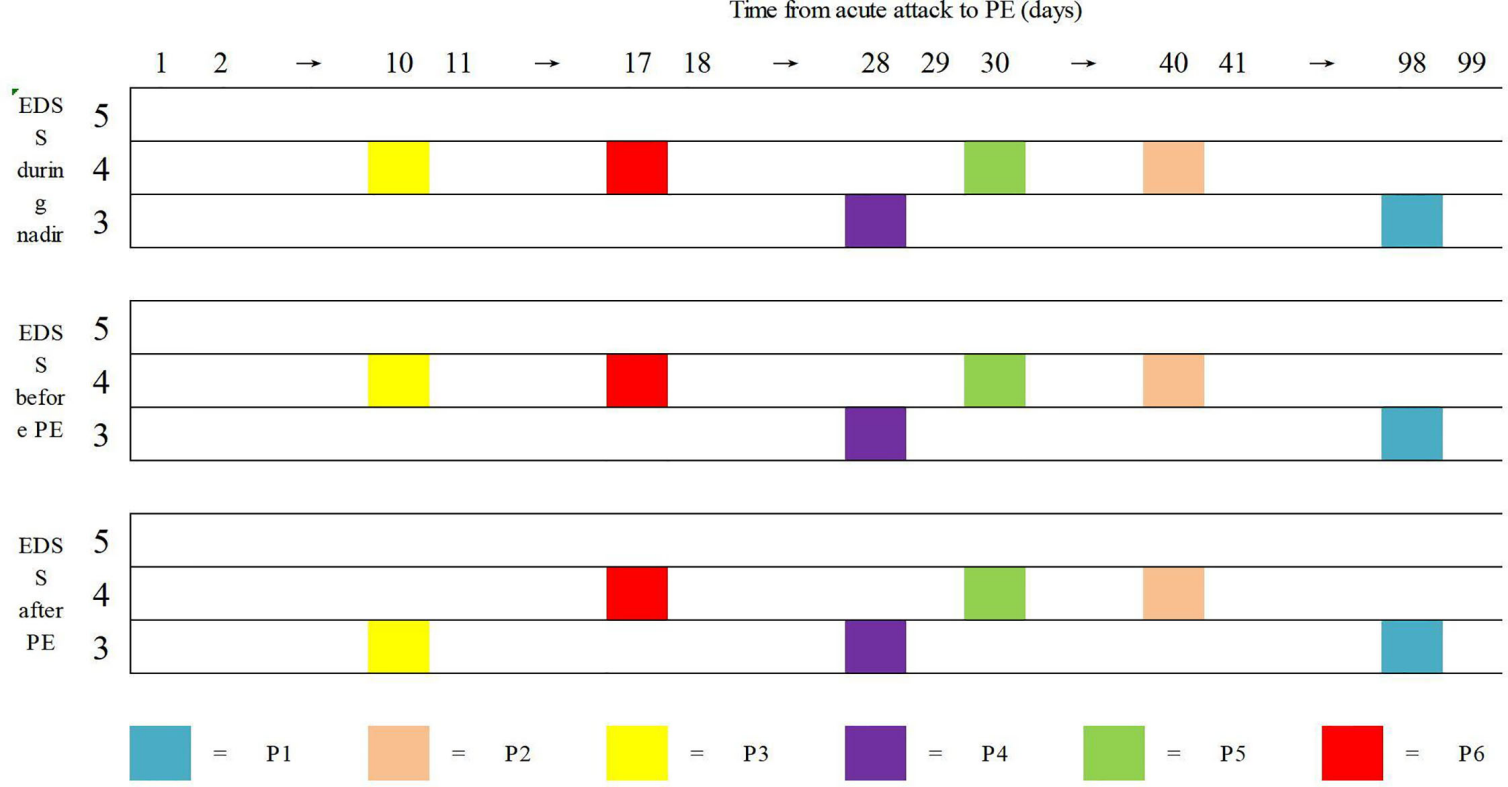
Data of Expanded Disability Status Scale (EDSS) of the six patients in three points. Horizontal coordinate is the time from acute attack to P.E. There were no clinical improvement after high dose IVMP. Only one patient (P3) had documented clinical improvement after PE procedures.

Regarding the efficiency of PE, only one patient (P3) exhibited clinical improvement after PE. Her left visual acuity increased from 0.06 to 0.6 (spectacle-corrected visual acuity was 1.0) and her EDSS score decreased from 4 to 3 points after PE. No improvement was observed in the other five patients after PE (**Figure 1**). Following plasma exchange, patients were administered RTX (P2, P3, P4, and P6), MMF (P1), and AZA (P5) to prevent relapse. At the last follow-up [3.71 (2.00–5.73) months] four patients (P2-P5) experienced no relapse following PE and immunosuppressive therapy, P1 suffered three relapses within 47 months of follow-up, and P6 suffered 1 relapse within 40 months of follow-up (**Table 2**).

Adverse events included rashes (P1, P3), acute non-occlusive thrombosis of the internal jugular vein (P1), and thrombocytopenia (P2) (**Table 3**).

**Table 3:** Adverse events associated with PE procedure.

NO.	P1	P2	P3	P4	P5	P6
hypotension	No	No	No	No	No	No
nausea	No	No	No	No	No	No
metabolic alkalosisi	No	No	No	No	No	No
hypocalcemia	No	No	No	No	No	No
hypofibrinogenemia	No	No	No	No	No	No
thrombocytopenia	No	Yes	No	No	No	No
acute non-occlusive thrombosis of an internal jugular vein	Yes	No	No	No	No	No
rash	Yes	No	Yes	No	No	No
anaphylaxis	No	No	No	No	No	No
transfusion-related acute lung injury	No	No	No	No	No	No
bacterial infection	No	No	No	No	No	No
other	No	No	No	No	No	No

## Discussion

NMOSD is rare among children, making up to 3–5% of all cases, with a higher prevalence in females than in males (16). The median age at onset in Chinese children is reported to be 14 years (range 7–17) (17). In our study, all 6 patients were female and the median age of onset was 11 years (range 8–13), which is consistent with the results of previous studies. Patients with AQP4-IgG have different clinical features, AQP4-IgG-NMOSD have worse treatment outcome, more attacks, worse prognosis than MOG-IgG-NMOSD (3), therefore antibody testing for AQP4-IgG and MOG-IgG should be done after the first attack which have positive implications for the diagnosis and therapy of NMOSD.

The water channel protein AQP4 is widely distributed in astrocytic foot processes at the blood-brain barrier and is particularly concentrated in the grey matter, periaqueductal, and periventricular regions of the spinal cord (4). Once bound to the extracellular domain of the AQP4 receptor, AQP4-IgG causes complement- and cell-mediated damage to astrocytes (18), which ultimately leads to the loss of support to surrounding cells (19). This is followed by granulocyte infiltration, oligodendrocyte injury, and demyelination.

In NMOSD, disabilities can occur due to the inflammatory damage caused during acute attacks, therefore, treatment during the acute attack is crucial to improve the prognosis (20). IVMP is typically the first-line treatment for acute attacks. The timing of IVMP initiation is crucial since there is a higher risk of poor recovery if the treatment is initiated seven days after the commencement (21). PE is the most widely used rescue treatment in patients who fail to recover substantially following IVMP (10). From a pathophysiological perspective, the efficacy of PE is reasonable. After five cycles of PE, AQP4-IgG is eliminated in up to 85% of NMOSD patients (22). Several studies have demonstrated that PE during the acute attack benefits patients with NMOSD (10, 23, 24). However, these studies mainly included adults, and only a few studies have evaluated the safety and effectiveness of PE in children with NMOSD (12, 25).

It has been demonstrated that patients who receive PE alone or in combination with IVMP have a better prognosis than those who receive IVMP alone (26). Initiating PE therapy within five days of onset may have better clinical outcomes, while the recovery rate of patients who received PE therapy after more than ten days of onset is roughly similar to that of NMOSD patients who were treated with steroids only (12, 27). In our study, only one patient (P3) had a 10-day delay in initiating PE therapy and the patient exhibited clinical improvement and a decrease in EDSS score after the therapy. Mickael et al.’s study showed an improved clinical benefit of early initiation of PE during severe attacks of NMOSD, but a complete improvement was only attained in 5%–20% of the patients treated with PLEX delay exceeding 10 days (27). In our study, the other five patients who received PE therapy ten days(range 17-98days) after the acute attack exhibited poor recovery, consist with previous study (27), the poor efficacy of PE therapy might be due to the delay in PE initiation after the acute attack. For the poor efficacy, another potential reason could be that the study subjects were no first-episode patients.

There is a broad consensus that the use of PE therapy in pediatric patients is more challenging due to the frequent vascular access issues, lesser blood volume, higher frequency of adverse effects, and the less cooperative nature of children (28). The adverse effects of PE observed in our study were consistent with those previously reported for PE in acute CNS demyelination (29). In our study, one patient received fewer PE sessions than initially planned due to severe thrombocytopenia, one patient developed rashes after the first PE cycle, and one patient developed rashes during PE and acute non-occlusive thrombosis of the internal jugular vein after five cycles of PE. PE has a potential risk for clinically significant adverse effects that should be discussed with the child and family before initiating the therapy and weighed against potential benefits.

Our study analyzed six cases only, therefore, the conclusions should be taken cautiously. Moreover, data were missing in some clinical records because of the retrospective nature of the study. Furthermore, EDSS scores might have been misclassified because they were calculated retrospectively. However, since the rater was blinded to the EDSS analysis timing, the bias had no significant effect on the conclusion.

## Conclusion

We describe our experience of PE treatment in six children with NMOSD. As a rescue therapy for severe and corticosteroid-unresponsive pediatric NMOSD, the timing of PE initiation is crucial. Moreover, PE has a potential risk for clinically significant adverse effects that we should be concerned about and explore potential preventive measures.

## Data availability statement

The original contributions presented in the study are included in the article/supplementary material. Further inquiries can be directed to the corresponding author.

## Ethics statement

The studies involving human participants were reviewed and approved by Ethics Committee of First Medical Center of PLA General Hospital. Written informed consent from the participants’ legal guardian/next of kin was not required to participate in this study in accordance with the national legislation and the institutional requirements.

## Author contributions

ZL, LW and GY wrote the first draft of this manuscript. LW and GY contributed to study conception and design. All authors contributed to the article and approved the submitted version.
